# A mixed methods evaluation of yoga as a fall prevention strategy for older people in India

**DOI:** 10.1186/s40814-018-0264-x

**Published:** 2018-05-01

**Authors:** Lisa Keay, Devarsetty Praveen, Abdul Salam, K. V. Rajasekhar, Anne Tiedemann, Vimala Thomas, Jagnoor Jagnoor, Cathie Sherrington, Rebecca Q. Ivers

**Affiliations:** 1 Injury Division, The George Institute for Global Health Australia, The University of Sydney, Level 5,1 King Street Newtown, Sydney, 2042 Australia; 2 Population Health Division, The George Institute for Global Health India, 301, Second Floor, ANR Centre, Road No 1, Banjara Hills, Hyderabad, 500034 India; 3 Cardiovascular Division, The George Institute for Global Health India, 301, Second Floor, ANR Centre, Road No 1, Banjara Hills, Hyderabad, 500034 India; 4 Centre for Physical Fitness and Sports Sciences, University of Hyderabad, Rao Rd, CUC, Gachibowli, Hyderabad, Telangana 500046 India; 5 Musculoskeletal Health Sydney, Sydney, Sydney School of Public Health, The University of Sydney, King George V Building, Sydney, 2042 Australia; 6 Osmania Medical College, Hyderabad, India; 7 Medical Education Office, State Government of Andhra Pradesh, Hyderabad, India

**Keywords:** Fall prevention, India, Mixed methods, Older people, Yoga

## Abstract

**Background:**

Falls are an emerging public health issue in India, with the impact set to rise as the population ages. We sought to evaluate the acceptability, feasibility and likely impact of a yoga-based program aimed at improving balance and mobility for older residents in urban India.

**Methods:**

Fifty local residents aged 60 years and older were recruited from urban Hyderabad, Andhra Pradesh. They were invited to attend a 1-h yoga class, twice weekly for 3 months. Mixed methods were used to evaluate the acceptability and feasibility (qualitative) and likely impact (quantitative). Two focus groups and eight interviews with participants were conducted to evaluate the acceptability and feasibility of a yoga program. Thematic analysis was conducted in context of perceptions, barriers and benefits of yoga participation and fall ascertainment. Physical performance using the Short Physical Performance Battery, fear of falling, blood pressure and weight loss were measured before and after the program.

**Results:**

The interviews and focus groups provided insights into the preferred format for classes, including session times, level of supervision and location. Improvements were seen in the Short Falls Efficacy Scale-International (Short FES-I (15.9 ± 4.0 vs 13.8 ± 2.1 s, *p* = 0.002)), the number of steps taken in the timed 4-m walk (T4MW (9.0 ± 1.8 vs 8.6 ± 1.8, *p* = 0.04)), Short FES-I scores (9.4 ± 2.9 vs 8.6 ± 2.9, *p* = 0.02) and weight (63.8 ± 12.4 vs 62.1 ± 11.6, *p* = 0.004) were lower. No changes were seen in standing balance, blood pressure or T4MW time.

**Conclusion:**

Yoga was well accepted and resulted in improved ability to rise from a chair, weight loss, increased step length and reduced fear of falling. These results provide impetus for further research evaluating yoga as a fall prevention strategy in India.

## Background

Fall-related injuries are a major cause of mortality and morbidity globally; it is estimated that 75% of fall injuries occur in low- and middle-income countries [1]. Previous investigations have identified falls in older people in India as a major public health issue [2, 3] but intervention studies have been neglected to date. In rural Andhra Pradesh, a community survey found that falls were the second most common cause of fatal injuries and the leading cause of non-fatal injuries across all age groups [4]. A community survey of adults over 60 years of age in urban Chandigarh and rural Haryana found that 52% of the 200 survey participants had fallen in the last year and that fractures occurred in one in every five falls [5].

Several approaches have been shown to reduce the risk of falls in older people including exercise, cataract surgery, medication reduction and home safety interventions [6]; however, none of these approaches have been evaluated in low-income settings. Exercise can reduce the risk of falls by up to 35% [7, 8] and also offers many other health benefits. Such fall prevention programs include the Otago home-based exercise program [9] and Tai Chi [10]; however, it is not certain that these programs would be acceptable to older people in India.

Yoga aims to enhance the physical, mental and spiritual aspects of health and involves practising a series of postures (asanas) and breathing exercises. Yoga has its origins in India and is likely to be a culturally acceptable mode for an exercise-based fall prevention program. A previous review identified five randomised controlled trials of yoga for physical fitness and function in older adults and reported improvements in leg strength, balance and mobility [11]. A randomised controlled trial found that a 12-week Iyengar yoga program improved balance and mobility in older adults in Australia [12]. This yoga program also improved functional measures previously identified as valid predictors of fall risk [13]. This led the authors to conclude that Iyengar yoga shows promise as a fall prevention intervention. An evaluation of an intensive yoga program in a residential aged care home in India found improvements in gait and balance [14].

Since yoga has been shown to improve balance and mobility in older adults in several settings [11, 12, 14], our primary aim was to assess the acceptability, feasibility and likely impact of yoga as a means to prevent falls through improving balance and mobility in a community setting in urban India. Such pilot investigations provide valuable guidance ahead of larger trials. Secondary outcomes included physical performance, fear of falling, blood pressure and weight loss. The feasibility of reporting falls in this setting was also investigated.

## Methods

This mixed methods pilot study of a 3-month yoga program involved 50 participants. Physical measures of strength and balance, body mass index and blood measure were assessed before and after participation to estimate the likely impact of the yoga program.

Available participants joined focus groups or were interviewed individually in the last week of the program to gain insights into acceptability and feasibility. All interviews and focus groups were facilitated by a trained male research assistant fluent in the local language and familiar to the participants through organising yoga sessions. The protocol was approved by the Osmania Medical College Human Research Ethics Committee, and written informed consent was obtained from each participant.

### Participants and recruitment method

All participants were residents of Harajpenta, the urban field practice area of Osmania Medical College. Field workers have an established relationship with the community and recruited participants by personal invitation. People were eligible to participate if they were aged 60 years and over and able to attend yoga classes at the local site. People were excluded from participation if they were not able to walk independently unaided or with a walking aid or had general health, hearing and speech impairments.

### Yoga intervention program

Participants were recruited in March 2012 and invited to attend two, 1-h yoga classes per week for 3 months at a temple neighbouring the urban health centre. The classes were taught by a yoga teacher with a Master’s of Science, Yoga Science and with experience in teaching yoga to older people. The yoga program had a fall prevention focus and emphasised standing poses which challenged balance [8, 12]. Physical supports could be used as required. Each yoga class included 5–10 min of warm-up, the main sequence of postures for 50 min (Table 1), breathing exercises for 5 min and meditation for 5 min.

**Table 1:** Sequence of postures and description of each

Posture name	Description
Uttana Padasana	Raised leg pose
Chakra Padasana	Leg rotation
Pada Sanchalanasana	Only forward movement cycling
Jhulana Lurhakanasana	Rocking and rolling
Shava Udharkarssanasana	Spinal twist
Naukasana	Boat pose
Namaskarasana	Salutation pose
Vyagrasana	Tiger pose
Ushtrasana	Camel pose
Hasta Uttanasana	Hand-raising pose
Tadasana	Tree pose
Tiryaka tadasana	Swaying palm tree pose
Tiryaka kati chakrasana	Swaying waist rotating pose
Dhruta Utkatasana	Dynamic energy pose
Dwikonasana	Double angle pose
Bhujangasana	Cobra pose
Artha shalabasana	Half locust pose
Eka Pada pranamasana	One legged prayer pose
Eka padasana	One foot pose
Sarvangasana	Shoulder stand pose

### Study measures

Trained research assistants administered a short interview and The Short Physical Performance Battery to each participant in their own homes at baseline and after the 3-month program. The interview collected social and demographic information, general health status, measured fear of falling with the Short Falls Efficacy Scales-International (Short FES-I) [15] and history of previous falls. The Short Physical Performance Battery [16], involves standing in six positions (feet apart with and without assistance, feet together side by side stand, semi-tandem stand, full-tandem stand and a one-leg balance test) each evaluated for up to 10 s, a timed 4-m walk at fast pace and a timed five-repetition sit to stand test. These tests have previously been shown to be associated with risk of falling [13] and were included to provide information about the likely effectiveness of the yoga program to prevent falls. Blood pressure (average of three measurements), weight and height were also measured.

Falls were reported prospectively to field test telephone follow-ups to document falls in this setting.

### Focus groups/interviews

In the final week of classes, all participants were invited to remain after the class for a focus group discussion, conducted separately for women and men. A focus group discussion is suitable when groups of people are likely to have common concerns or shared experiences [17]; however, these would not capture the views of those participants who had dropped out of the program. Therefore, up to eight participants not in attendance at the final week of classes were invited to take part in semi-structured in-person interviews. These guided discussions were moderated by a trained research assistant, observed by one of the investigators (AS) and covered three sets of topics using a corresponding set of prompts: perceptions of the yoga program, perceived benefits of yoga and understanding fall injury/reporting falls (Table 2). Each focus group and interview was audio-recorded, transcribed verbatim and translated into English. The transcripts were reviewed by bilingual research assistants who attended the sessions to verify accuracy.

**Table 2:** Focus group and semi-structured interview discussion guide

General topics	Prompts
Perceptions of the yoga program	• What were your initial thoughts about doing yoga? • What did you enjoy/not enjoy? • Were you comfortable during lessons? • Was the venue and time of day acceptable? • Was there anything that stopped you going to classes? • What would make classes easier to attend?
Benefits of yoga	• Did you feel any benefits from yoga? • Did you notice any changes after doing the yoga classes? • Were there any negative effects? • Would you recommend yoga to friends and family? • What type of people is best suited to yoga?
Understanding of falls injury and reporting of falls	• Are you worried about falling? • Is it easy to answer about whether you have fallen at classes or by phone? • How often would we need to ask about falls?

### Data analyses

Directed content analysis is a targeted approach which is suitable when there are a priori issues and previous research in this area [18]. Therefore, this analytic approach was chosen to investigate the acceptability and feasibility of yoga as a fall prevention program in this setting. The discussion guide included questions about the perceptions, barriers and benefits of the yoga program but alluded to concepts about environmental constraints, skills and ability, and norms in line with the Integrative Model of Behavioural Prediction [19].

After familiarisation with the transcripts, the text was coded against the integrative framework [19] which recognises that while intention is central to the likelihood that a behaviour will occur, there are environmental factors and individual skills and abilities that can influence behaviour. While the analysis was largely deductive, any new themes related to participation in the program that emerged from the data were also considered. Further, as the practical considerations for reporting falls had not been investigated in this setting, we sought information about the best way to report falls. At the second stage, the investigative team discussed the categorised content and produced themes which answer the question about acceptability and feasibility of a yoga-based fall prevention program in this setting. Excerpts from the transcripts were chosen to illustrate the themes. Analyses were conducted in NVivo 9 (QSR International).

Changes in the outcome measures from the baseline to the conclusion of the yoga program were analysed using paired *t* tests. All analyses were conducted in SAS Enterprise Guide Version 7.1 (Cary, NC, USA). Allowing for 10% drop-outs and using published distributions of measures of physical performance in another population [20], a sample size of 50 was required to detect a 4-s change in the five times sit to stand test (standard deviation (SD) = 9) or test of standing balance (SD = 9) and 0.1 m/s change in gait speed (SD = 0.25) [21]. The study was not powered to measure the impact of yoga on falls.

## Results

The study’s recruitment target of 50 was met after inviting 86 potential participants (participation rate 58%). Amongst those who declined to join the study, 11 stated that they could not attend early morning yoga classes due to other commitments (mostly household work), 8 declined due to poor health and 17 were not interested. Amongst the group who were not interested, two stated that their family members were opposed to them attending and three stated that they had religious restrictions or did not believe in yoga.

### Participant characteristics

The mean age of the participants was 64 years (SD = 6, range 60–81); they predominantly followed Hinduism (48/50, 96%) and the majority spoke Telugu at home (48/50, 96%). Twenty-nine (58%) of the 50 participants were women, and the most frequently reported occupations were home duties (26/50, 52%) or retired/unemployed (11/50, 22%). Other occupations included manual workers (6), owner of a business or farm (2), office workers (3) and professionals (2). Most of the housing in which participants lived had fixed walls and roofs of durable construction (34/50, 68% pucca housing) with around one third living in housing with roofs of other materials (16/50, 32% semi-pucca). Approximately two thirds of the participants needed to use the stairs in the house each day, and two thirds had a bathroom outside of the home. The participants were married (28/50, 56%) or widowed (22/50, 44%) and typically lived in extended nuclear families with an average of five people in the home. The average annual household income was US$300 per year (INR14, 830 ± 17,056, range 2000 to 120,000) and 15/50 (30%) had primary education or no education.

Diabetes (18/50, 36%) and hypertension (27/50, 54%) were the most prevalent medical conditions with heart attack, angina, stroke, peripheral vascular disease and high cholesterol reported in < 12% of the participants. Six participants (12%) reported impaired distance vision, defined by trouble recognising a person across the street. A total of 36/50 people (75%) were currently taking medications prescribed to them by a health care provider, and approximately half of the men (10/21, 48%) and none of the women reported drinking alcohol. Eight participants (16%) reported having had a fall in the previous 12 months, and one participant reported two falls during this time.

### Intervention

We held 27 yoga classes at 6:30 A.M. taught by one or two instructors, with average duration of 62 min (range 45 min to 1 h and 15 min). The attendance varied between 18 and 31 participants per class. The average number of classes attended was 12 (44%, standard deviation (SD) = 11). Nine participants (18%) did not attend any classes, and 17 participants (34%) attended at least 75% of the classes. No falls were reported during the 3-month study period, and no adverse events as a result of the yoga intervention were reported.

### Qualitative data: focus groups and interviews

Fifteen women and eight men participated in the post-intervention focus group discussions which had duration of 47 and 43 min respectively. An additional eight participants were interviewed individually. The participants enjoyed the yoga program and stated that they would recommend yoga to others. Yoga was compared to *English exercise* by several participants and considered to be superior. In an interview, it was stated: ‘In the yoga exercises every part will be activated. In other exercises like English exercise it will not be like that. In this every part has to be moved.’ Yoga was perceived as a local rather than a foreign discipline, and therefore, an accepted and favoured form of exercise. The theme of ‘yoga is a holistic and preferred exercise regime’ supported the theory that attitudes can contribute to intention consistent with the integrated theory of behaviour change.

This program was designed to enhance balance and strength, and several statements from participants indicated that they found improvements of this type: ‘after doing yoga I am feeling far better and I can freely walk and move my legs freely, I can have balance.’ Another stated that a fear of falling deterred him from attending yoga classes though the male focus group discussion suggested that fear of falling was generally alleviated by the yoga program. The female focus group participants reported being able to walk further and gaining balance whereas the men talked more frequently about gains in fitness. The confidence to complete the program and gain perceived benefits are summarised in the theme of ‘yoga to gain independent mobility’ and where present was derived from a strong sense of self-efficacy where an individual has belief in their control over a process and perceived power to participate [19].

The participants reported numerous other beliefs about the benefits of yoga but control of blood pressure (13 citations) and diabetes (11 citations) were the most commonly cited benefits. Other benefits were for gains in mental health: ‘mind active’, ‘good condition’, ‘concentration increased’, ‘eliminates laziness’ and general enjoyment of group activities. Several stated they had lost weight, and there were a range of other self-perceived benefits such as ‘helps blood flow’, reductions in ‘back pain, knee pain’ and less symptoms of ‘numbness’ and ‘nervous weakness’. The theme of ‘yoga for healthy ageing’ was supported by the wide range of benefits to cardiovascular, metabolic and mental health raised by the study participants. The consensus was that yoga is appropriate and a normal activity [19] for older people and can promote health, safety and prevent future illness: ‘if we do yoga, we will live longer life’.

Much of the discussions addressed environmental factors which can influence participation. There was a view that practising yoga at home was not appropriate due to lack of adequate space, distractions, need for guidance and trouble remembering the sequence of postures. Only one participant who had not attended the sessions had a preference for doing yoga at home with a television for instruction. Holding the classes early in the morning was considered convenient due to other commitments later in the day and a preference for early rising. Other recommendations included having an audio system so instructions could be heard in a large venue and providing more than one teacher or an attendant for better instruction.

The most common reason given for non-attendance was illness, but there were also situations where the participants were not available due to family commitments for funerals, caring duties, pilgrimages or religious holidays. Amongst those who discontinued, there was some discomfort about doing yoga at the venue as it was in public view. The environmental considerations can be summarised under the concepts of ‘privacy’ and ‘convenience’. The comments relating to being able to hear instruction relate to the individual skills and abilities in the integrative model and highlight how sensory impediments detracts from ability to participate successfully in large group classes.

All participants were aware of the risks and serious consequences of having a fall in older age. Many related anecdotes of the devastating consequences for friends and family who had fallen and suffered injuries themselves. In order to undertake further research on fall prevention in India, valid methods for fall reporting need to be established. Participants did not report concerns about their ability to recall having a fall; however, there were some suggestions that people may not want to report a fall or could be confused about what researchers define as a fall. One participant stated: ‘they won’t reveal it even if they have fallen down due to insult.’ There was a preference for personal discussion rather than phone calls about whether the participant has fallen.

### Quantitative data: measures of impact

Three participants were lost to follow-up so paired comparisons were completed for 47 participants (Table 3). After the yoga program, the study participants performed significantly faster in the five times sit to stand test (15.9 s ± 4.0 vs 13.8 ± 2.1, *p* = 0.002). The time taken to complete the 4-m walk did not change significantly after the program, but participants took less steps to walk the 4-m distance (9.0 ± 1.8 vs 8.6 ± 2.9, *p* = 0.04). There was no change in performance on the test of standing balance, although all but one participant (49/50, 98%) attempted the highest level of difficulty on this task at baseline (standing on one leg for 10 s) and 28/50 (56%) could hold the position for the required 10 s, demonstrating a possible performance ceiling effect in this population. The mean score on the Short FES-I questionnaire was lower at follow-up, indicating significantly less concern about falling (9.4 ± 2.9 vs 8.6 ± 2.9, *p* = 0.02). Mean body weight was also reduced at follow-up (63.8 ± 12.4 vs 62.1 ± 11.6 kg, *p* = 0.004); however, there was no change in blood pressure.

**Table 3:** Mean (standard deviation) performance in outcome measures from baseline to post-intervention (3 months) and statistical significance of the change

	Baseline	3 months	*P* value*
Sit to stand (seconds)	15.9 ± 4.0	13.8 ± 2.1	0.002
Standing balance (seconds)^#^	57 ± 4	58 ± 4	0.70
Gait:			
Timed 4-m walk (seconds)	5.8 ± 1.7	5.5 ± 1.8	0.2
Number of steps in 4 m	9.0 ± 1.8	8.6 ± 1.8	0.04
Short Falls Efficacy Scale-International	9.4 ± 2.9	8.6 ± 2.9	0.02
Weight	63.8 ± 12.4	62.1 ± 11.6	0.004
Systolic blood pressure	141 ± 23	139 ± 21	0.5
Diastolic blood pressure	82 ± 13	83 ± 12	0.5

^#^Six positions assessed for up to 10 s: feet apart with assistance, feet apart without assistance, feet together side by side stand, semi-tandem stand, full-tandem stand and one-leg stand

*Paired *t* test for *n* = 47 who completed the baseline and follow-up assessments

## Discussion

In this pilot study, we evaluated a yoga-based program aimed at reducing fall risk by improving balance and mobility amongst a group of older people in an urban Indian community. This study contributes valuable information about acceptability and feasibility of yoga in this context. A central concept emerging from the focus groups and interviews was that yoga has a wide range of benefits beyond improvements in balance and mobility—‘yoga for health ageing’. A meta-analysis of the benefits of yoga for older people found that there were benefits for self-reported health, aerobic fitness and strength, supporting the perceptions found in the current study [22]. It was encouraging that we documented weight loss. Lifestyle interventions which include programs to improve physical activity may be the best way to reduce the burden of chronic disease in the long-term, particularly in low-resource settings. These findings suggest that yoga is a holistic approach to healthy ageing and the other health benefits, such as diabetes and hypertension status, should be evaluated in addition to falls.

Yoga may be best positioned as a program for ‘healthy ageing’ rather than simply for fall prevention, and this could alleviate the challenge of engaging with older people who have multiple competing health priorities [23]. Research into the perceptions of fall prevention programs in Europe has also found that ‘falls prevention’ per se is perceived by older people as patronising and distressing [24]. Presenting yoga programs as having multiple benefits may alleviate this concern.

We found that the venue and time of day were important considerations for participants. Ideally, yoga would be provided in a location with good access for older members of the community, preferably a closed location with no distractions where older people would feel comfortable participating in yoga. Early mornings were favoured in this population as it allowed time for other activities later in the day. Of note, many participants recommended yoga to their peers and there were some instances where members of the community who were not involved in the study attended the classes.

The qualitative data indicated that yoga was a familiar form of exercise in this population group, considered holistic and culturally acceptable. The response rate to our direct invitation to participate suggested a moderately positive attitude to yoga at the outset though attendance was relatively low. Investigations of the reasons for not joining the study or non-attendance at classes did not reveal any systematic problems with the program. Non-participation was usually due to poor health or cultural obligations, such as religious festivals, and family or carer responsibilities, which took older members of the community away from their home for long periods. Attendance at our program was lower than in other studies, and this is a limitation of our study as dose (the amount of yoga completed) may have been insufficient. Nyman published a review of participation in and engagement with fall prevention interventions in community settings with data from 99 trials, 31 of which were class-based exercise [25]. Our attendance rates were modest compared to other fall prevention trials which were reported at 83% dropping to 76% over 24 months. More flexibility in session times or an alternative venue which was not open to the public view may have increased attendance but this cannot be determined from this analysis.

Though participation was modest, we identified improvements in functional balance and mobility outcomes and fear of falling after 12 weeks. These findings are in agreement with studies in other settings, including randomised controlled studies that demonstrated improvements in physical measures of balance after a yoga program [12, 14, 26, 27]. Yoga may be an effective fall prevention strategy as some of these measures have been shown to be related to fall risk [13, 15]. While these results indicate likely benefits to physical function, the effectiveness of this program needs to be evaluated in an appropriately powered randomised trial.

We also investigated logistical issues like the most appropriate method to prospectively report falls. Use of monthly fall calendars is recommended as best practice [28]; however, low levels of literacy, lack of personal space for maintaining calendar records and failings in the postal system make this approach difficult in India. We field tested phone calls and in-person prompts for falls. The participants expressed a preference for personal contact, concerns over understanding the definition of a fall and bias in reporting.

To our knowledge, this is the first pilot study evaluating a fall prevention program for older people living in a community in India. It is a limitation of the study that our population was confined to an urban community. The response may have been different in rural areas, amongst other religious groups and people from a higher socio-economic background. We also did not have a control group, and the improvements may reflect a learning effect rather than a difference due to the yoga program, though findings from controlled trials in other settings suggest otherwise [12].

## Conclusions

This study has identified some important preliminary indicators of the viability of yoga as a fall prevention strategy for older people in India. The findings lend confidence to this approach and provide guidance for future more comprehensive evaluations, including randomised controlled trials.

## Abbreviations

Short FES-I: Short Falls Efficacy Scale-International
T4MW: Timed 4-m walk
